# Supplementary material for: Ca2+-mediated higher-order assembly of heterodimers in amino acid transport system b0,+ biogenesis and cystinuria
Source: Nat Commun. 2022 May 16;13:2708. doi: 10.1038/s41467-022-30293-9 (PMC9110406; doi:10.1038/s41467-022-30293-9)

Supplementary Information

Ca^{2+}-mediated higher-order assembly of heterodimers in amino acid transport system $b^{0,+}$ biogenesis and cystinuria

Yongchan Lee[1,2*†], Pattama Wiriyasermkul[3,4*], Pornparn Kongpracha[3,4], Satomi Moriyama[4], Deryck J. Mills[1‡], Werner Kühlbrandt[1] and Shushi Nagamori[3,4†].

[1] Department of Structural Biology, Max Planck Institute of Biophysics, 60438, Frankfurt, Germany.
[2] Graduate School of Medical Life Science, Yokohama City University, Kanagawa, 230-0043, Japan.
[3] Department of Laboratory Medicine, The Jikei University School of Medicine, Tokyo, 105-8461, Japan.
[4] Department of Collaborative Research for Bio-Molecular Dynamics, Nara Medical University, Nara, 634-8521, Japan.

[*] These authors contributed equally.
[‡] Deceased July 7, 2020.
[†] Corresponding authors. Email: yongchan.lee@biophys.mpg.de (Y.L.) or snagamori@nagamori-lab.jp (S.N.).

This file includes:
Supplementary Figures 1 – 16
Supplementary Tables 1 – 2
Source Data of Supplementary Figures

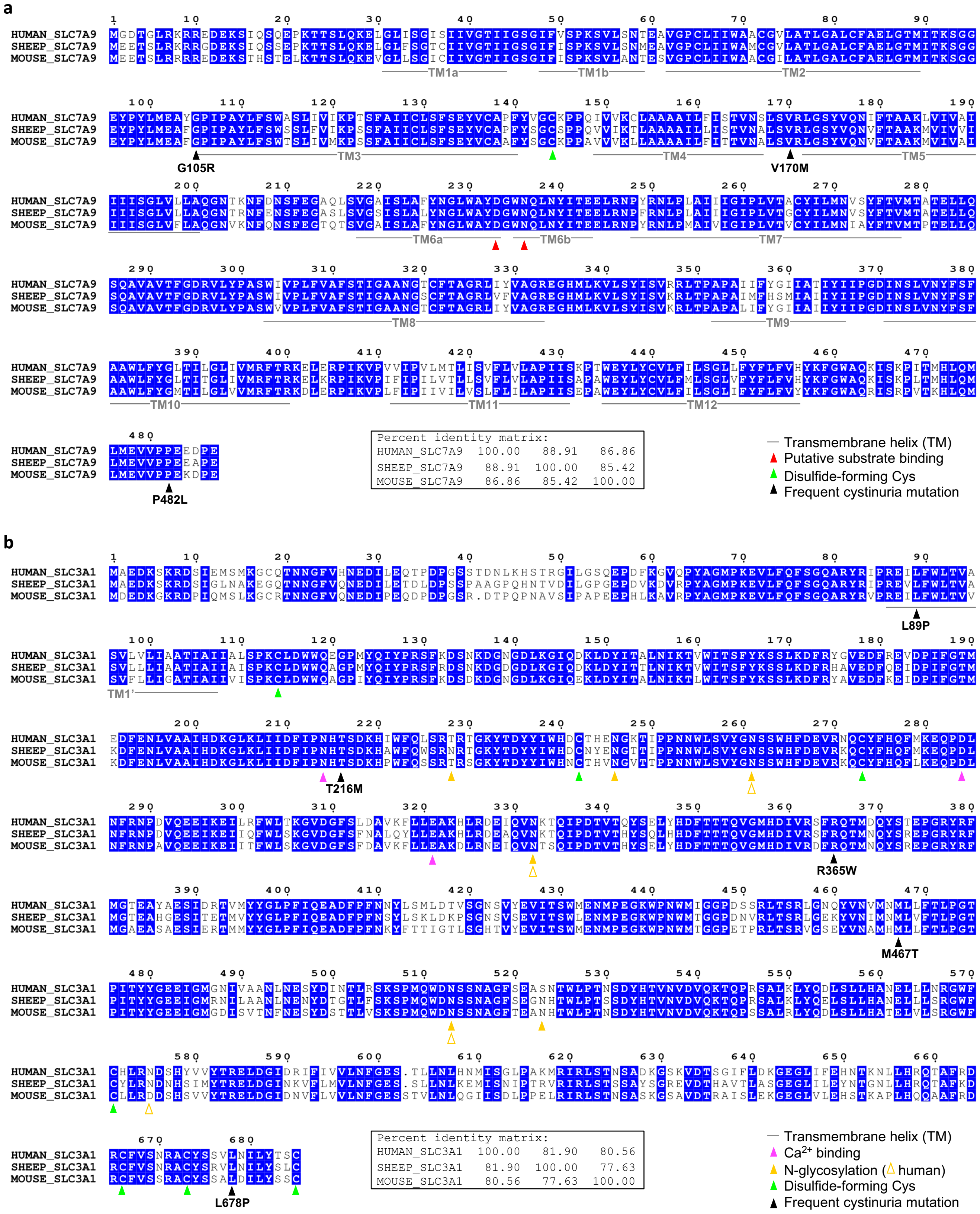

Supplementary Figure 1 | Sequence alignments of $b^{0,+}$AT and rBAT

a) Multiple sequence alignment of human (HUMAN_SLC7A9), ovine (SHEEP_SLC7A9), and murine $b^{0,+}$AT (MOUSE_SLC7A9). Alignments were generated by Clustal Omega and formatted by ESPript. Conserved residues are colored blue. The small inset shows the sequence identities (%) between each pair of sequences.

b) Multiple sequence alignment of human (HUMAN_SLC3A1), ovine (SHEEP_SLC3A1), and murine rBAT (MOUSE_SLC3A1).

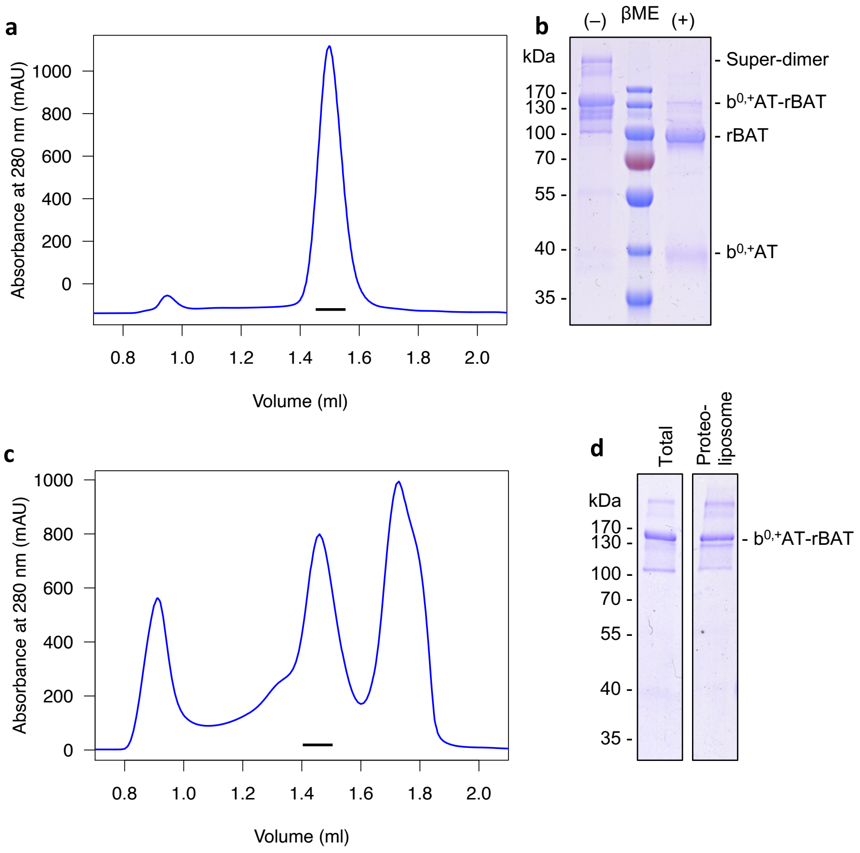

Supplementary Figure 2 | Sample preparation of $b^{0,+}$AT–rBAT

a) Size-exclusion chromatography (SEC) profile of purified ovine $b^{0,+}$AT–rBAT complex. The monodisperse peak marked by a black bar was used for cryo-EM and biochemical analyses. "mAU" stands for "milli-absorbance units".

b) SDS-PAGE analysis of $b^{0,+}$AT–rBAT. The purified complex was subjected to non-reducing (left) and reducing SDS-PAGE (right).

c) SEC profile of nanodisc-reconstituted $b^{0,+}$AT–rBAT. Peak fractions marked by a black bar were used for cryo-EM analysis. The lower molecular-mass peak is excess MSP1E3D1 scaffold protein.

d) Reconstitution of $b^{0,+}$AT–rBAT into proteoliposomes. Samples were run on non-reducing SDS-PAGE before and after liposome reconstitution, indicating a reconstitution efficiency of nearly 100%.

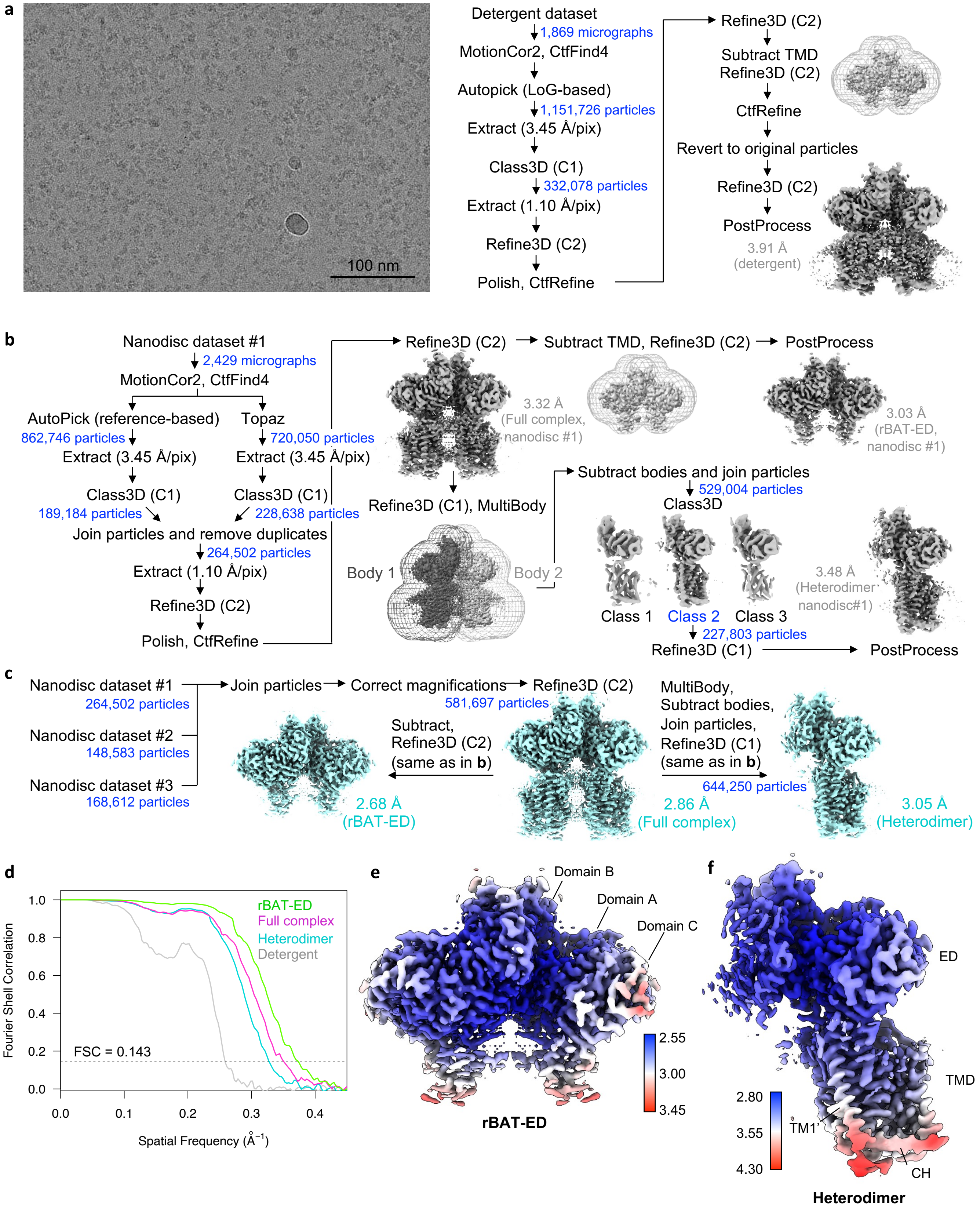

Supplementary Figure 3 | Single-particle data processing of b$^{0,+}$AT–rBAT

a) A representative micrograph and processing workflow of the detergent dataset.

b) Processing workflow of nanodisc dataset #1.

c) Merging procedure for nanodisc datasets #1–#3, acquired on three different electron microscopes. Merged data yielded better resolution than individual sets, after correcting for small errors in pixel sizes. Final maps used for model building are colored in cyan. Resolutions for half-map FSC = 0.143 are shown.

d) Gold-standard FSC curves calculated for individual maps.

e) Local resolution of rBAT-ED dimer.

f) Local resolution of heterodimer subcomplex.

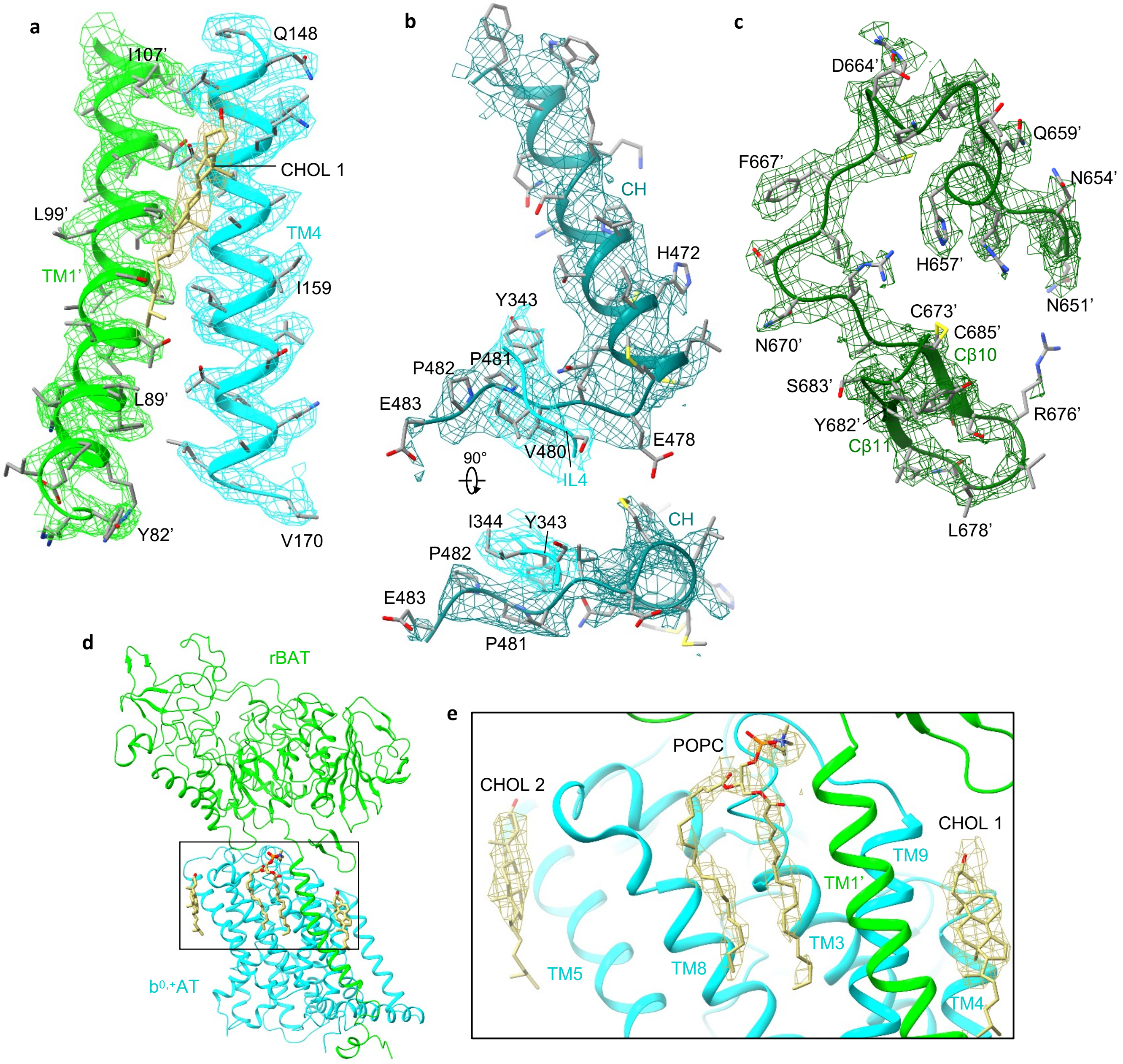

Supplementary Figure 4 | Cryo-EM maps in key regions of b$^{0,+}$AT–rBAT

a) TM1’–TM4 interface with a bound cholesterol (CHOL1). Leu89’ is highlighted.

b) C-terminal helix (CH) of b$^{0,+}$AT. The Val-Pro-Pro motif interacts with intracellular loop 4 (IL4).

c) C-terminal peptide of rBAT.

d) Three lipids modelled into the density.

e) Cryo-EM map of the three lipids, derived from the heterodimer map (**Supplementary Fig. 3f**).

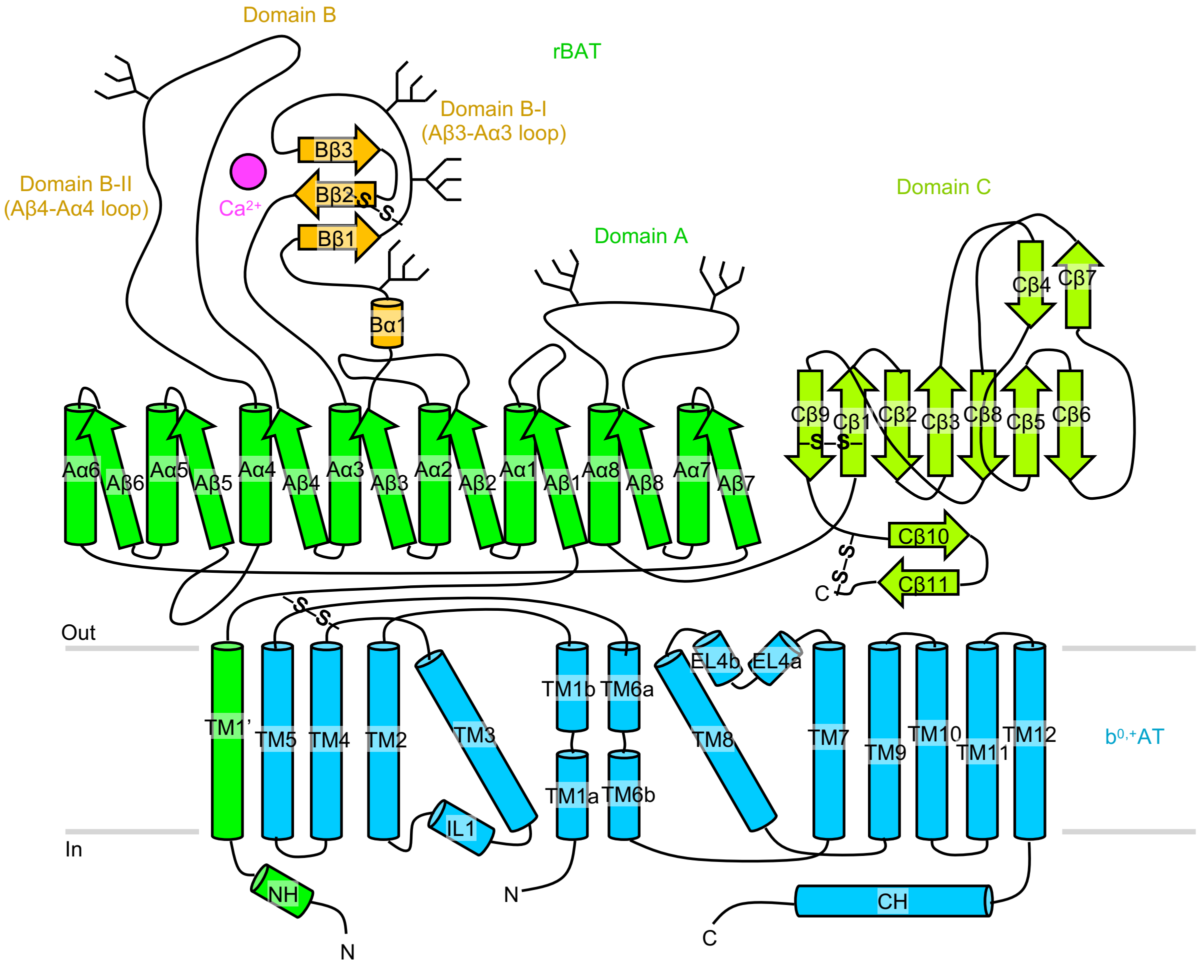

Supplementary Figure 5 | Domain architecture of ovine $b^{0,+}$AT–rBAT

Domain diagram of $b^{0,+}$AT–rBAT, with focus on secondary structure elements, subdomains and post-translational modification (N-linked glycosylation, disulfide bonds and Ca^{2+} binding). rBAT consists of subdomains A, B and C, TM1', NH and the disordered N-terminal region. Domain B is composed of two loops designated as domain B-I and B-II. $b^{0,+}$AT consists of twelve transmembrane helices (TM1–TM12), two short helices in extracellular loop 4 (EL4a and EL4b), one helix in the intracellular loop 1 (IL1) and the C-terminal helix (CH). The N terminal ~50 residues are disordered in $b^{0,+}$AT.

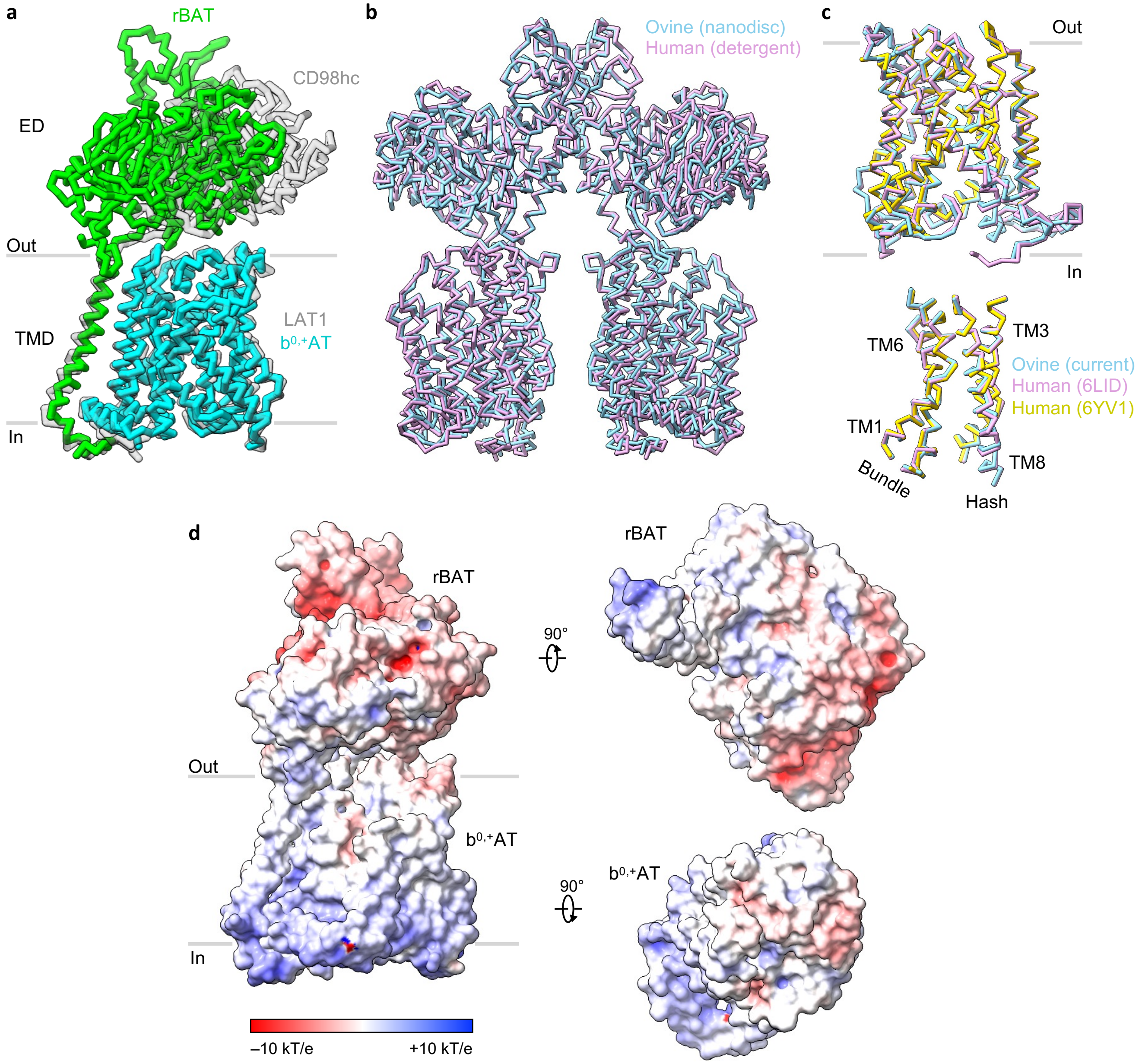

Supplementary Figure 6 | Assembly mechanism of b$^{0,+}$AT–rBAT

a) Structural difference between b$^{0,+}$AT–rBAT and LAT1–CD98hc. Structures were superimposed on the TMD. The rBAT ectodomain shows a shift towards TM1' compared to CD98hc, due to the shorter linker between TM1' and ED *(9)*.

b) Hetero-tetrameric assembly of ovine and human b$^{0,+}$AT–rBAT (PDB 6LID). Structures were superimposed by the Matchmaker tool in ChimeraX. The two structures are almost identical. The RMSD between 2,152 aligned atom pairs is 1.350 Å.

c) Superposition of ovine b$^{0,+}$AT (this study) with two published structures of human b$^{0,+}$AT (PDB IDs: 6LID and 6YV1). The lower panel depicts the relative positions of the rocking bundle (TM1 and TM6) to the hash domain (TM3 and TM8), showing that all three structures are in the same inward-facing conformations.

d) Electrostatic potentials for the b$^{0,+}$AT–rBAT heterodimer or separate subunits. Electrostatic potentials were calculated with APBS.

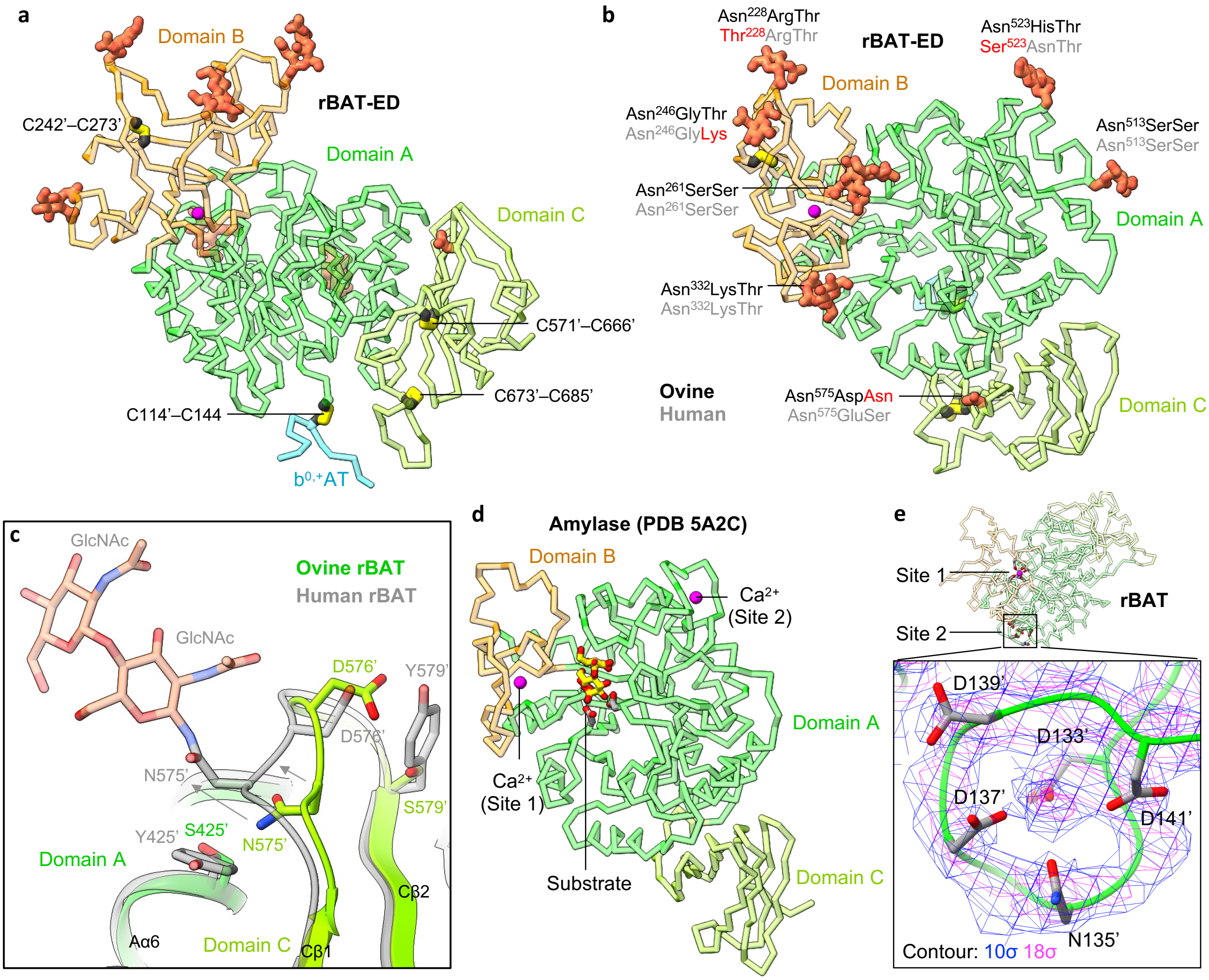

Supplementary Figure 7 | Disulfide bonds, N-linked glycans and metal-binding site of rBAT

a) Disulfide bonds of rBAT. The bond at C114'–C144 is formed across subunits (rBAT and $b^{0,+}$AT), whereas the other bonds are formed within the subunit.

b) N-linked glycans of ovine rBAT. The underlying glycosylation motif NX(T/S) is shown for each site. Human rBAT sequences are shown in gray for comparison. Motif-breaking amino acid substitutions are labeled red. Asn575' in ovine rBAT is not glycosylated, whereas the corresponding residue in human is glycosylated.

c) Structural differences around Asn575' in domain C. Asn575' of ovine rBAT is not glycosylated and resides within the β-sandwich fold of domain C. In human rBAT, the corresponding residue is glycosylated, protruding towards the extracellular solvent, with acetylglucosamine moieties (GlcNAc) interacting with domain A. The sequence comparison shows that ovine rBAT has small residues around Asn575', namely Ser425' and Ser579', whereas human rBAT has bulky residues Tyr425' and Tyr579', which would result in steric clashes.

d) Structure of *Anoxybacillus* α-amylase, which resembles that of the rBAT ectodomain and has high-affinity Ca^{2+}-binding sites *(25)* (PDB ID: 5A2C). Site 1 (called Ca1 in the cited literature) is at the domain A–B interface and site 2 (Ca2) is in domain A. Site 1 is close to the substrate, shown as a yellow stick model.

e) Weak non-protein density at site 2 of ovine rBAT. The map is contoured at 10 σ and 18 σ. A similar map of site 1 is shown in **Fig. 5a**. Note that this site 2 structurally resembles an EF-hand motif, although it has no strict sequence homology.

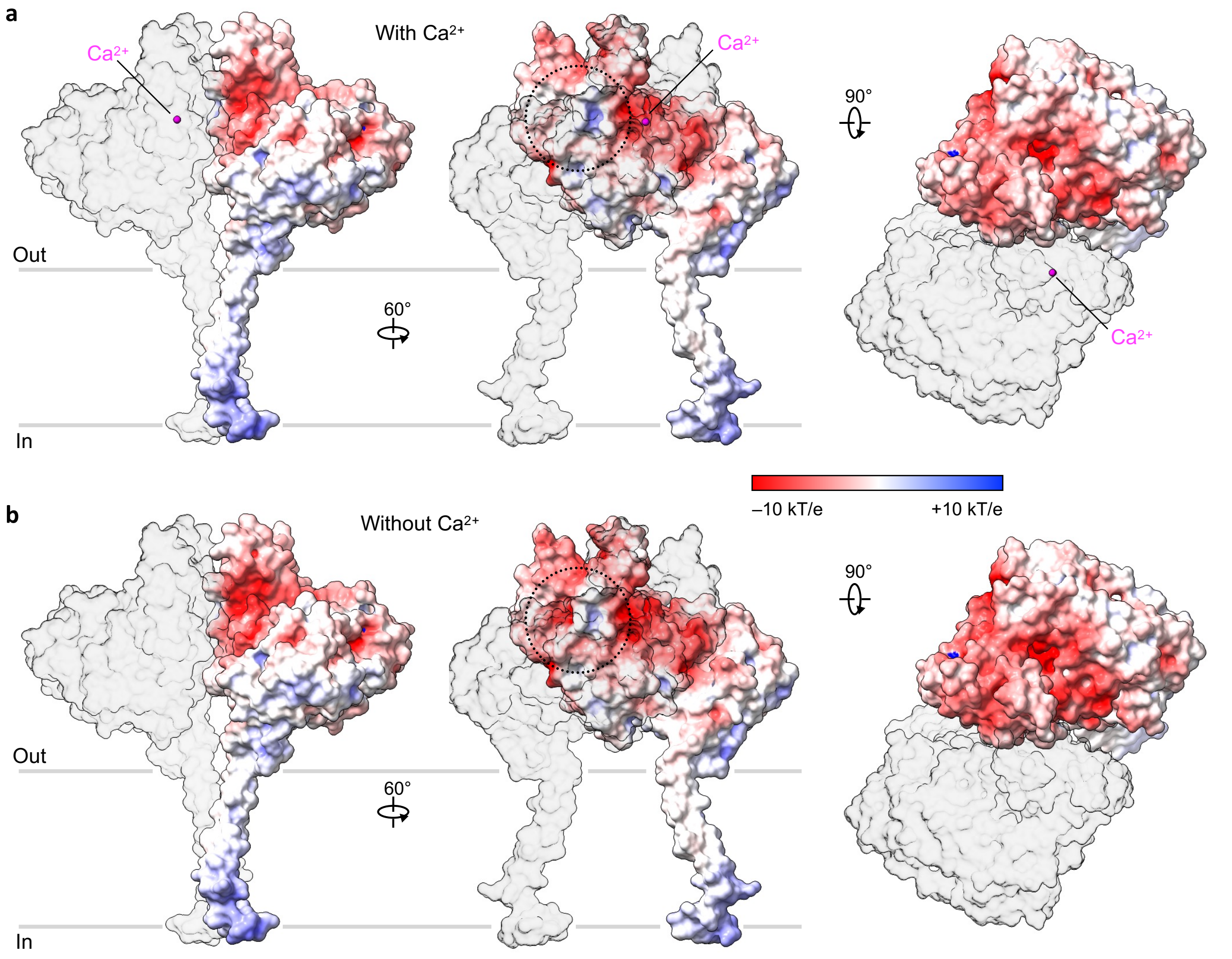

Supplementary Figure 8 | Surface electrostatic potential of rBAT

a, b) Electrostatic potential of rBAT with (**a**) and without Ca^{2+} (**b**). The surface patch near the Ca^{2+}-binding site shows a strong negative charge in the absence of Ca^{2+} (**b**, middle panel, dotted circle), whereas it is neutralized in the presence of Ca^{2+} (**a** , middle panel, dotted circle). Electrostatic potentials were calculated with APBS.

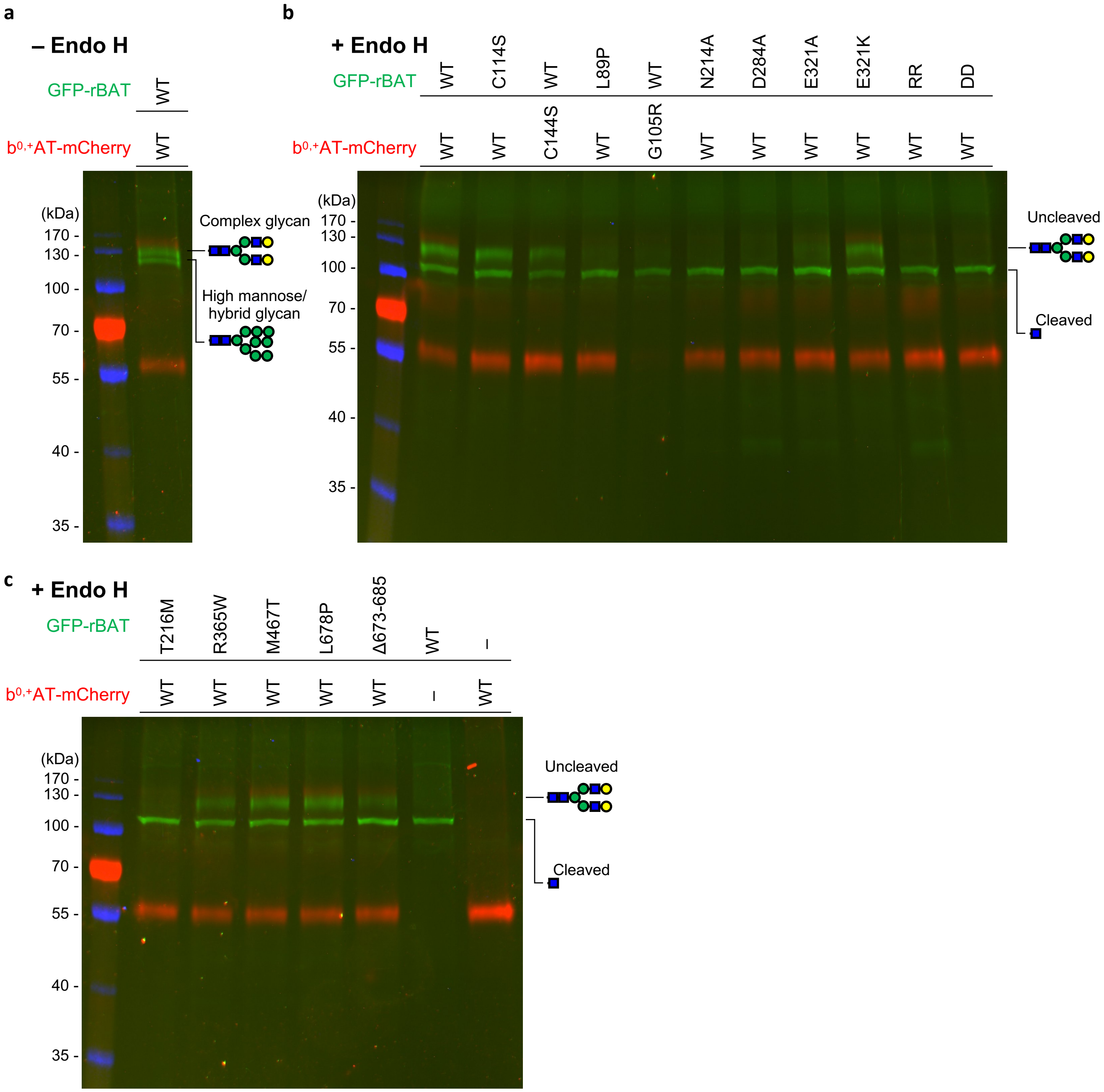

Supplementary Figure 9 | Endo H sensitivity assay

a) SDS-PAGE gel of cell lysate expressing GFP-rBAT and $b^{0,+}$AT-mCherry before Endo H treatment. The image is an overlay of three fluorescence channels for GFP (green, wavelength = 488 nm, exposure time = 120 sec), mCherry (red, 546 nm, 1440 sec) and the pre-stained molecular weight marker (blue, ex 680 nm, 2 sec). The GFP channel of the same gel is shown in **Fig. 5c**.

b, c) SDS-PAGE gels for rBAT and $b^{0,+}$AT, or their mutants, after Endo H treatment. GFP channels of the same gels are shown in **Figs. 5d,e and 8b**.

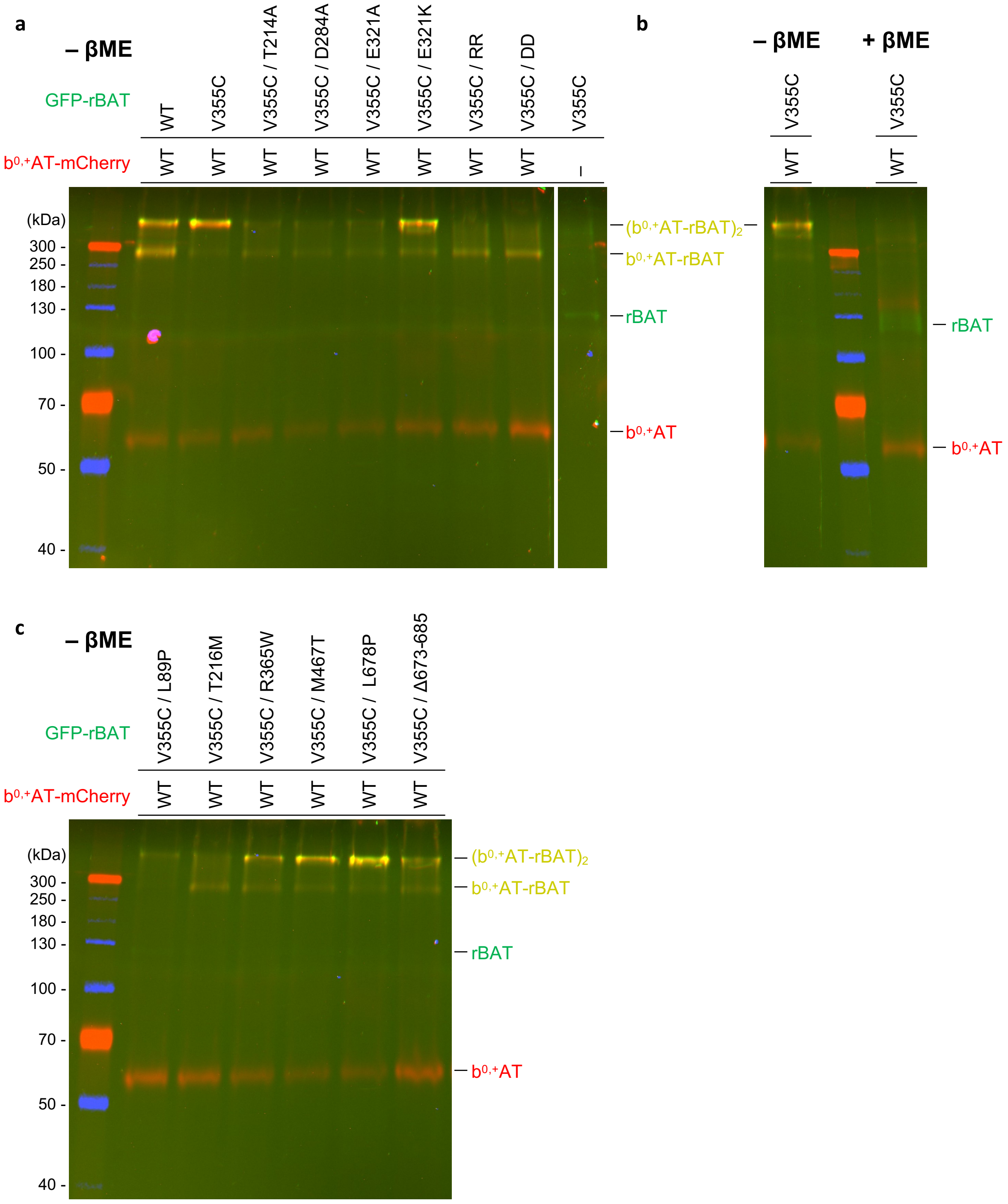

Supplementary Figure 10 | Site-specific disulfide cross-linking assay

a) Oxidizing SDS-PAGE for GFP-rBAT and $b^{0,+}$AT-mCherry, or their mutants, co-expressed in HeLa cells. The three fluorescence channels were imaged as in **Supplementary Fig. 9a–c**. The yellow bands thus correspond to the heteromeric complexes, of which the upper one is the super-dimeric $(b^{0,+}\text{AT-rBAT})_2$ complex, whereas the lower band is the $b^{0,+}$AT-rBAT dimeric complex. A cropped image of the same gel is shown in **Figs. 5g,h and 8a**.

b) Cross-linking validation. Upon addition of β-mercaptoethanol, the yellow band dissociates into two bands, each representing GFP-rBAT (green) or $b^{0,+}$AT-mCherry (red), confirming specific disulfide cross-linking. A cropped image of the same gel is shown in **Fig. 5i**.

c) Oxidizing SDS-PAGE for cystinuria mutants. A cropped image of the same gel is shown in **Fig. 8a**.

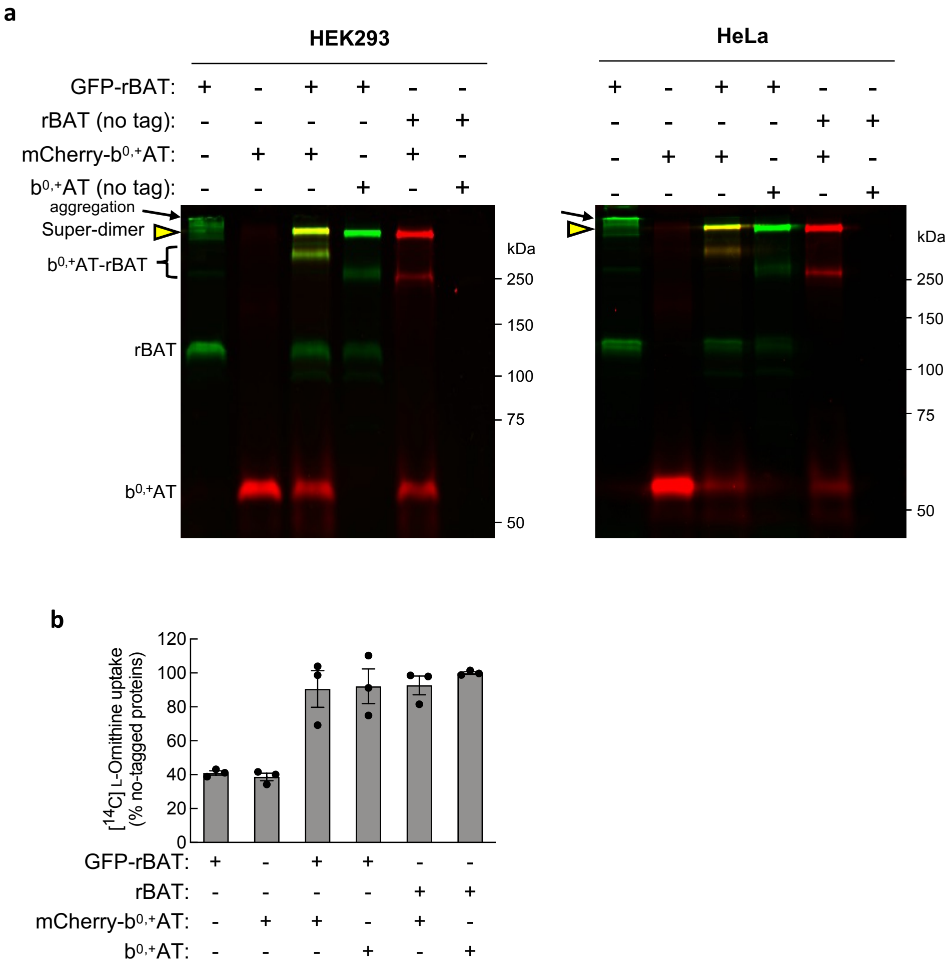

Supplementary Figure 11 | GFP and mCherry tags in b$^{0,+}$AT-rBAT do not alter the super-dimer formation or the protein functions.

a) HEK293 or HeLa cells were transfected with wild-type b$^{0,+}$AT and rBAT with different tags. The membrane fractions were subjected to 10% non-reducing SDS-PAGE. b$^{0,+}$AT and rBAT were visualized by fluorescent detection of mCherry (red) and GFP (green), respectively. Yellow is resulted from merge of mCherry and GFP signals. Both GFP and mCherry tags do not alter the presence of b$^{0,+}$AT-rBAT super-dimerization nor dimerization (different band sizes are due to tag sizes) in both types of cell lines.

b) Functional analysis of b$^{0,+}$AT and rBAT with different tags. HEK293 cells were transfected with wild-type b$^{0,+}$AT and rBAT with different tags. Functions of b$^{0,+}$AT-rBAT were evaluated by L-[^{14}C]ornithine uptake in Na^+-free buffer. The proteins with or without GFP or mCherry tags exhibit comparable transport functions. Values are mean ± SEM. n = 3 technical replicates.

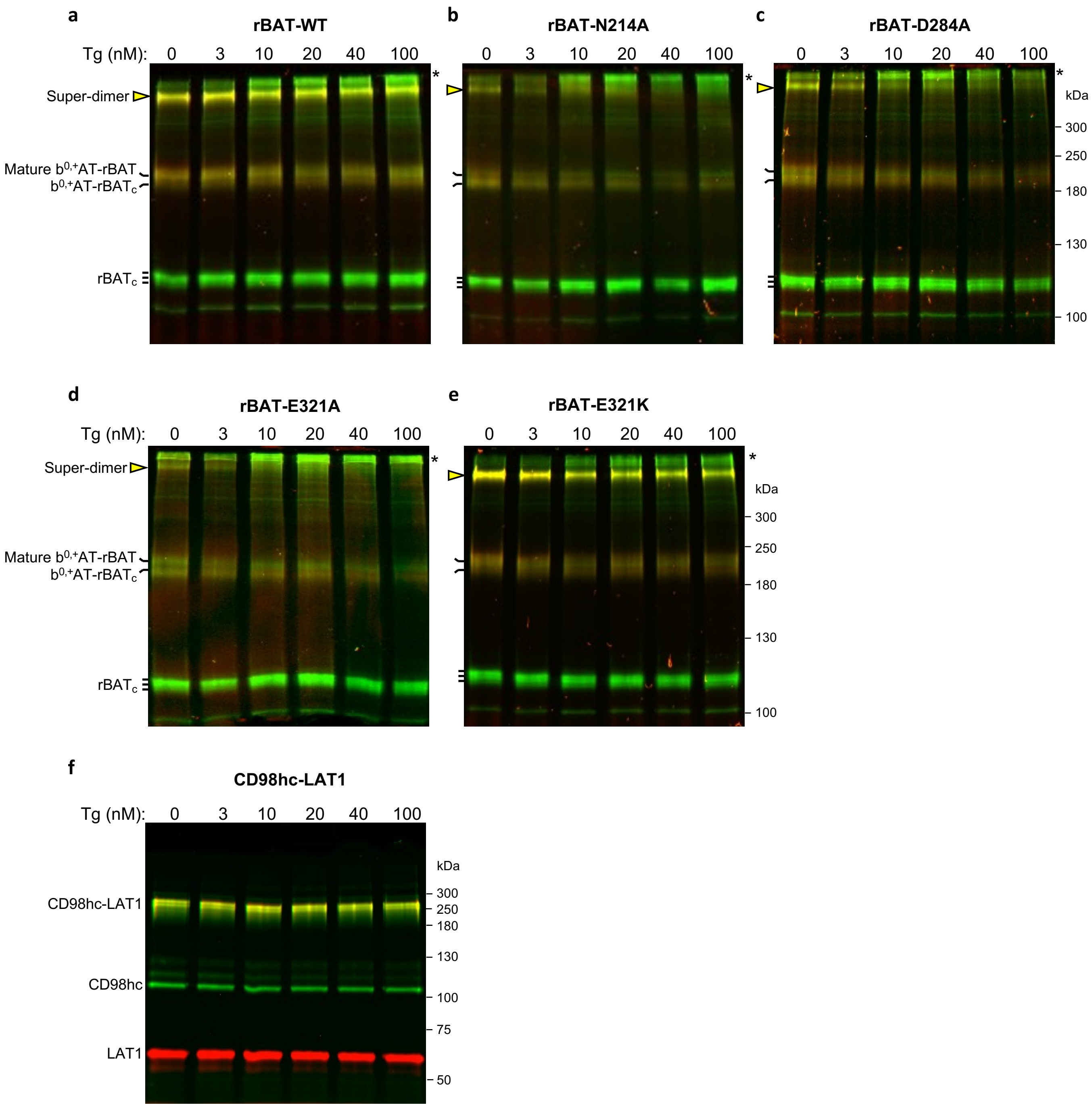

Supplementary Figure 12 | Effect of thapsigargin on the super-dimer formation

a–e) HeLa cells transfected with wild-type mCherry-$b^{0,+}$AT and wild-type **(a)** or mutated GFP-rBAT **(b-e)** were treated with thapsigargin (Tg) at different concentrations for 6 hrs. Membrane fractions were subjected to 7% non-reducing SDS-PAGE and the images display the overlayed GFP and mCherry fluorescence channels in which all images were captured at the same exposure time. Yellow thus represents the merge of mCherry and GFP. All rBAT constructs reveal super-dimer decrease by Tg concentration-dependent manner. Asterisk indicate protein aggregation at the top of polyacrylamide gels, which were gradually generated upon the increment of Tg concentration.

f) HeLa cells transfected with wild-type GFP-CD98hc and wild-type mCherry-LAT1, which does not form super-dimerization, were used as negative control. The membrane fraction was analyzed on 10% SDS-PAGE without reducing agent. Image displays the overlayed GFP and mCherry fluorescence channels.

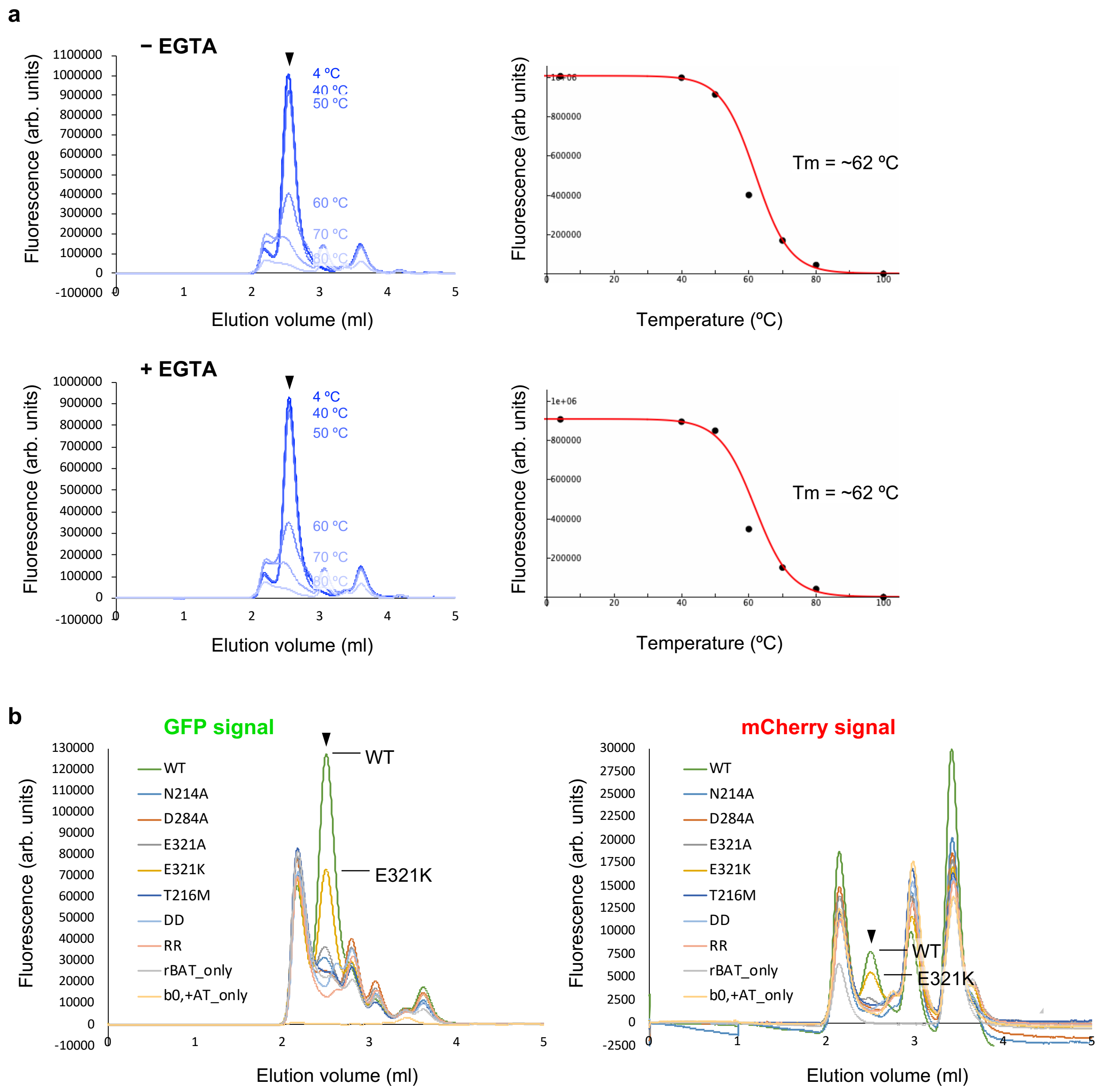

Supplementary Figure 13 | Stability of b$^{0,+}$AT–rBAT variants upon solubilization by detergent

a) Fluorescence-detection size exclusion chromatography was performed for wild-type GFP-rBAT and wild-type b$^{0,+}$AT-mCherry co-expressed in HEK293S GnTI^{-} cells. Elution profile was monitored in the presence or absence of 10 mM Ca^{2+}-chelating EGTA.

b) Fluorescence-detection size exclusion chromatography of b$^{0,+}$AT and rBAT-WT or several mutants. Elution profile was monitored by a dual-wavelength fluorescence detector, which simultaneously recorded GFP and mCherry signals. rBAT and E321K co-expressed with b$^{0,+}$AT showed a major peak at the expected elution volume (~2.5 ml; marked by black arrowheads), indicating that they are stable upon solubilization by detergent. By contrast, other variants were unstable and appeared as high-molecular weight aggregation (~2.1 ml; void volume) or dissociated into monomeric species (~2.8 ml in GFP and ~3.0 ml in mCherry recordings).

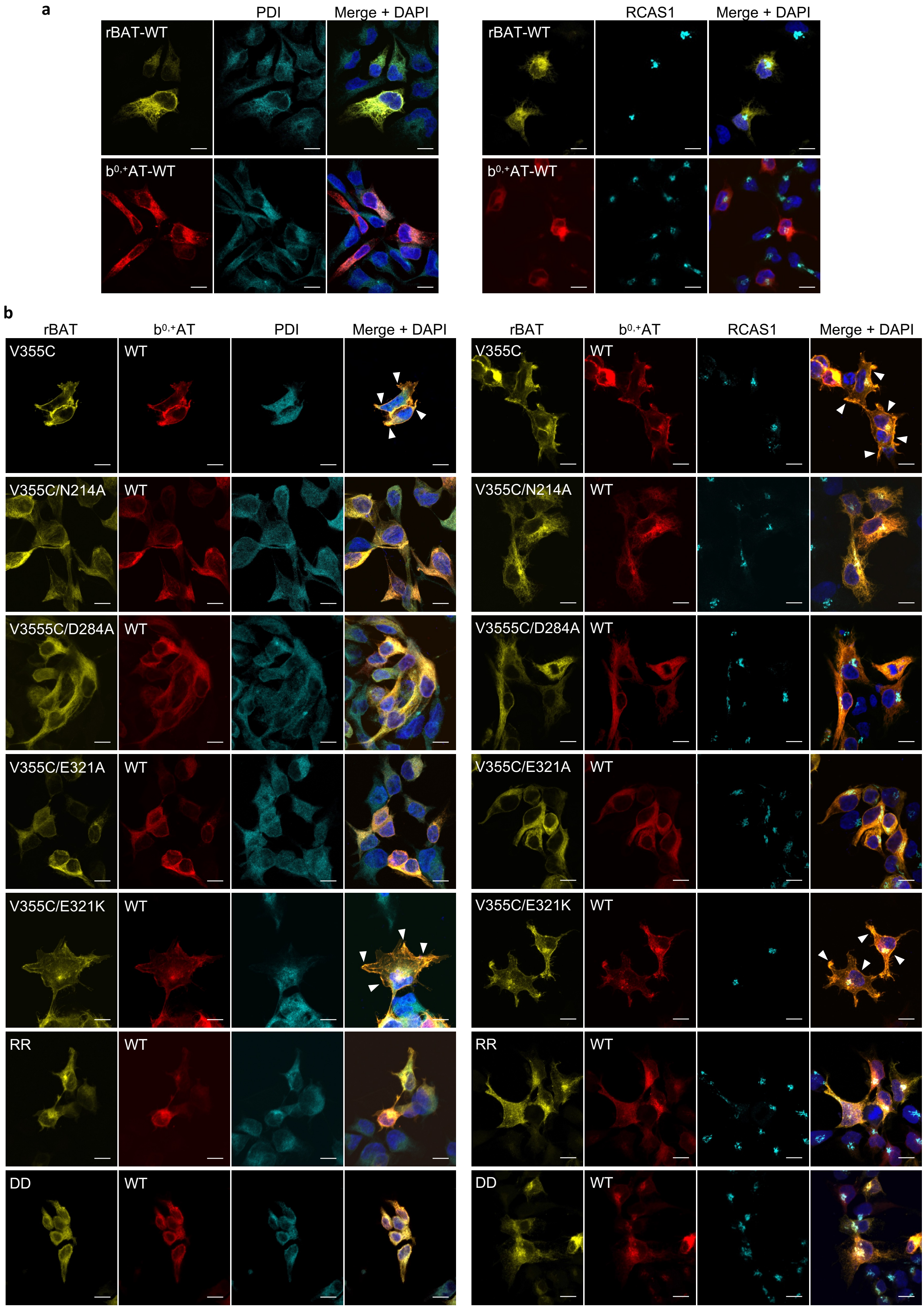

Supplementary Figure 14 | Fluorescent cell imaging

See next page for figure legends.

Supplementary Figure 14 | Fluorescent cell imaging

a) Fluorescence imaging of HeLa cells individually expressing rBAT-WT (GFP-tagged: yellow) or $b^{0,+}$AT-WT (mCherry-tagged: red) as negative controls. Staining with anti-PDI (cyan in the left panel) and anti-RCAS1 (cyan in the right panel) antibodies are used as the markers for ER and Golgi apparatus, respectively. Scale bar = 10 μm.

b) Immunofluorescence of HeLa cells expressing wild-type mCherry-$b^{0,+}$AT (red) and GFP-rBAT-V355C (yellow) or the double mutants as indicated. Staining with anti-PDI and anti-RCAS1 antibodies are used as markers for ER (cyan on the left panel) and Golgi apparatus (cyan on the right panel), respectively. Plasma membrane localization of $b^{0,+}$AT-rBAT are indicated in the white arrowheads. V355C/N214A, V355C/D284A, V355C/E321A, D349R/D359R (RR) and R326D/R362D (DD) are primarily retained in the ER while V355C/E321K are chiefly at the plasma membrane and Golgi apparatus. Scale bar = 10 μm.

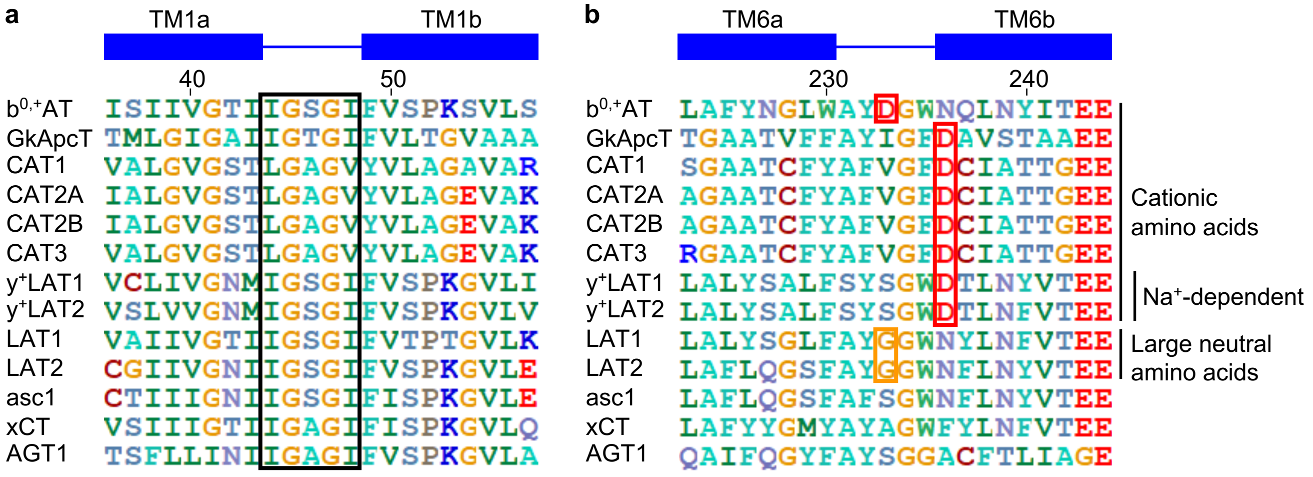

Supplementary Figure 15 | Sequence features of TM1 and TM6 in SLC7 transporters

a) Sequence alignment of TM1 in human SLC7 (SLC7A1–11, 13) and GkApcT. The unwound region between TM1a and TM1b, highlighted by a rectangle, is well-conserved across all SLC7 members. System $b^{0,+}$ member: $b^{0,+}$AT (SLC7A9). System y^{+} members: CAT1 (SLC7A1), CAT2A-B (SLC7A2) and CAT3 (SLC7A3). System y^{+}L members: y^{+}LAT1 (SLC7A7) and y^{+}LAT2 (SLC7A6). System L members: LAT1 (SLC7A5) and LAT2 (SLC7A8).

b) Sequence alignment of TM6 in human SLC7 (SLC7A1–11, 13) and GkApcT. Acidic residues implicated in cation recognition are outlined in red. Gly residues important in system L are are outlined in orange.

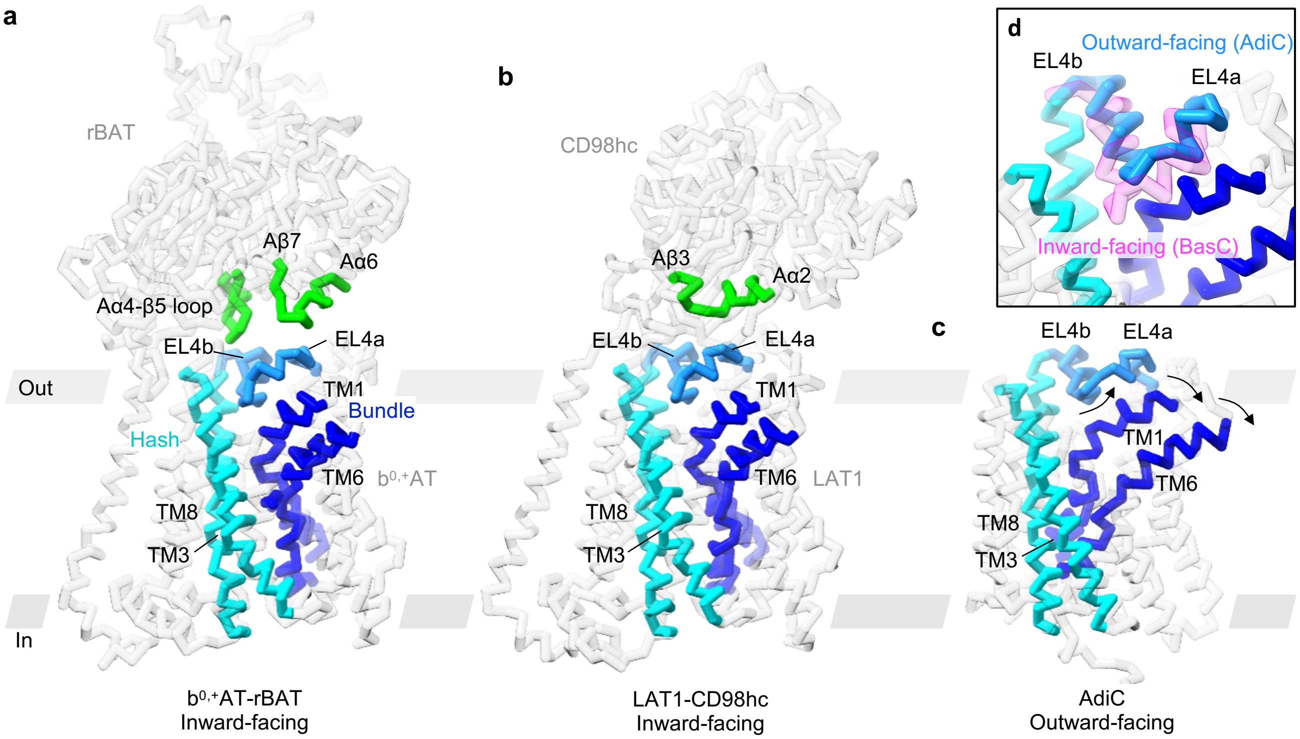

Supplementary Figure 16 | Possible mechanisms of SLC7 regulation by SLC3

a) Structure of $b^{0,+}$AT–rBAT, highlighting structural elements important for substrate translocation. Extracellular halves of TM1 and TM6 are close to TM3 and TM8, closing the extracellular gate. EL4a and EL4b act as a lid, stabilized by two loops of rBAT.

b) Inward-facing structure of LAT1–CD98hc. As in $b^{0,+}$AT, EL4a and EL4b of LAT1 form a lid above the extracellular gate, while interacting with a different CD98hc loop.

c) Structure of AdiC in the outward-facing conformation. Swinging movements of TM1, TM6, EL4a and EL4b define the opening of the extracellular gate and are depicted by arrows. Along with this movement, the extracellular halves of TM1 and TM6 dissociate from TM3 and TM8 to open the extracellular gate. EL4a and EL4b show concerted movements to widen the substrate pathway.

d) Close-up view of EL4a and EL4b in two bacterial SLC7 homologues. Comparison of the outward-facing structure of AdiC (cyan) and the inward-facing structure of BasC (pink) shows that EL4a and EL4b undergo sliding movements to enable a wide opening of the extracellular gate.

Supplementary Table 1 | Cryo-EM data collection

	Detergent	Nanodisc #1	Nanodisc #2	Nanodisc #3
**Data collection**				
Microscope	Titan Krios 2	Titan Krios 2	Titan Krios 1	Titan Krios 2
Camera	K3	K3	K3	Falcon III
Magnification	105,000	105,000	105,000	96,000
Voltage (kV)	300	300	300	300
Electron exposure (e^{-}/Å^2)	50.0	50.0	40.0	40.0
Defocus range (μm)	−1.4 to −2.5	−1.2 to −2.2	−1.0 to −2.0	−1.4 to −2.0
Calibrated pixel size (Å)	0.837	0.837	0.831	0.833
Initial particle images (no.)				
RELION / Topaz	1,151,726 / –	862,746 / 720,050	456,825 / 579,851	855,952 / –

Supplementary Table 2 | Data processing, model building and validation statistics

	Detergent	Nanodisc #1 – #3 merged		
		Full complex	rBAT ectodomain	Heterodimer
**Data processing**				
Final particle images (no.)	332,078	581,697	581,697	644,250
Final pixel size (Å)	1.09856	1.09856	1.09856	1.09856
Symmetry imposed	C2	C2	C2	C1
Map resolution (Å)				
Half map FSC = 0.143	3.91	2.86	2.68	3.05
Map sharpening *B* factor (Å^2)	–111.3	–63.1	–76.9	–85.1
Local resolution range (Å)			2.55–3.45	2.80–4.30
**Refinement**				
Initial model (PDB codes)			1UOK, 6AAV, 6IRS	1UOK, 6AAV, 6IRS
Refinement resolution (Å)			2.60	3.00
Model resolution (Å)				
Map-model FSC = 0.5			2.62	2.95
Model composition				
Non-hydrogen atoms			4,821[a]	8,817
Protein residues			575	1,078
No. ligands			GlcNAc: 8<br>Ca^{2+}: 1	GlcNAc: 8<br>Ca^{2+}: 1<br>POPC: 1<br>Cholesterol: 2
Average *B* factors (Å^2)				
Protein			50.24	72.85
Ligand			86.60	100.92
R.m.s. deviation				
Bond lengths (Å)			0.005	0.006
Bond angles (°)			0.672	0.718
**Validation**				
MolProbity score			2.25	2.46
Clashscore			5.36[b]	7.61
Rotamer outliers (%)			6.53	8.17
Cβ outliers (%)			0.00	0.00
CaBLAM outliers (%)			3.68	2.62
Ramachandran plot				
Favored (%)			95.11	94.88
Allowed (%)			4.71	5.03
Outliers (%)			0.17	0.09

[a] for one asymmetric unit.
[b] Clashscore is calculated for a C2-expanded homodimer to take homomeric interfaces into account.

Source Data of Supplementary Figures.

Supplementary Fig. 2b: uncropped image

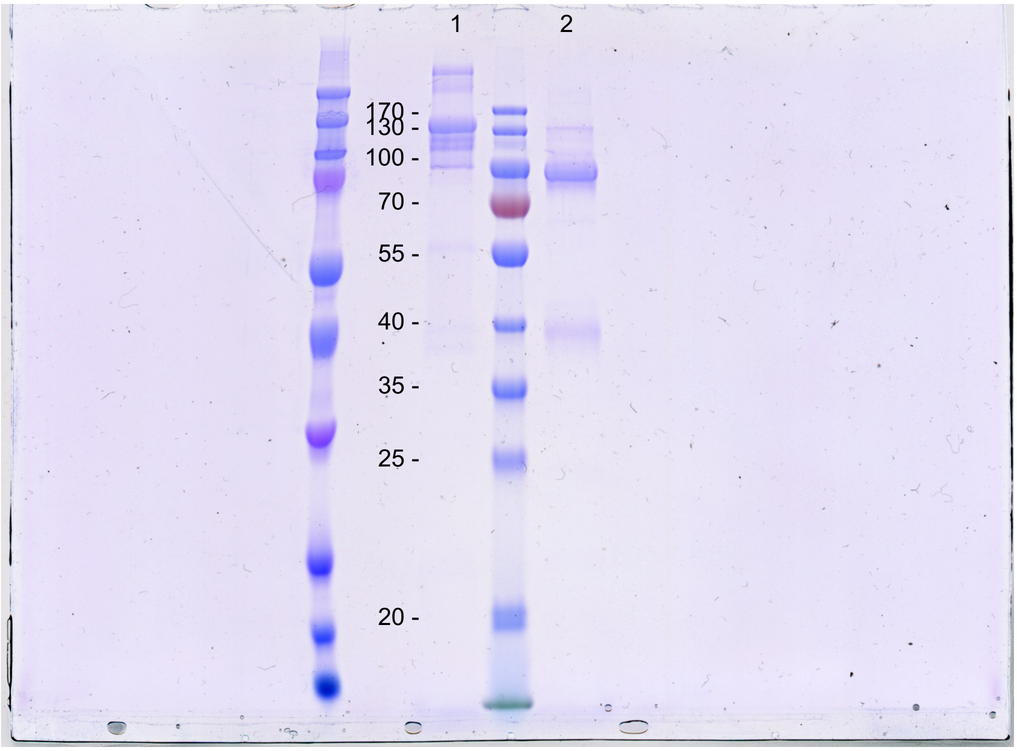

Supplementary Fig. 2d: uncropped image

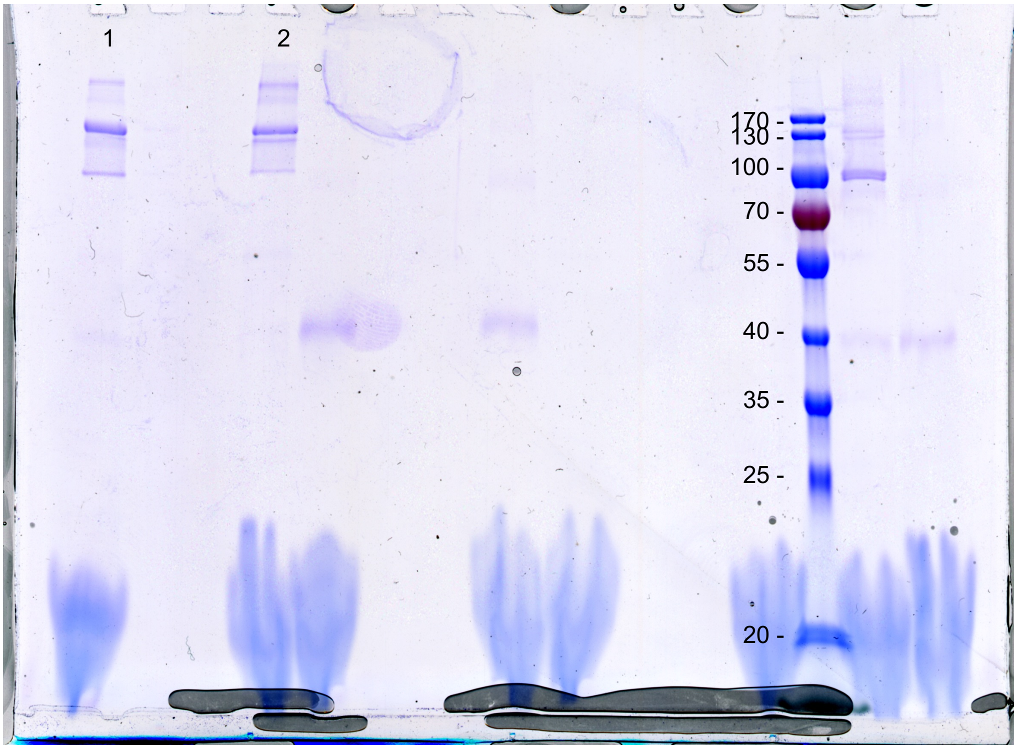

Supplementary Fig. 9: uncropped images
(GFP channels of the same gel are also shown in Figs. 5 and 8)

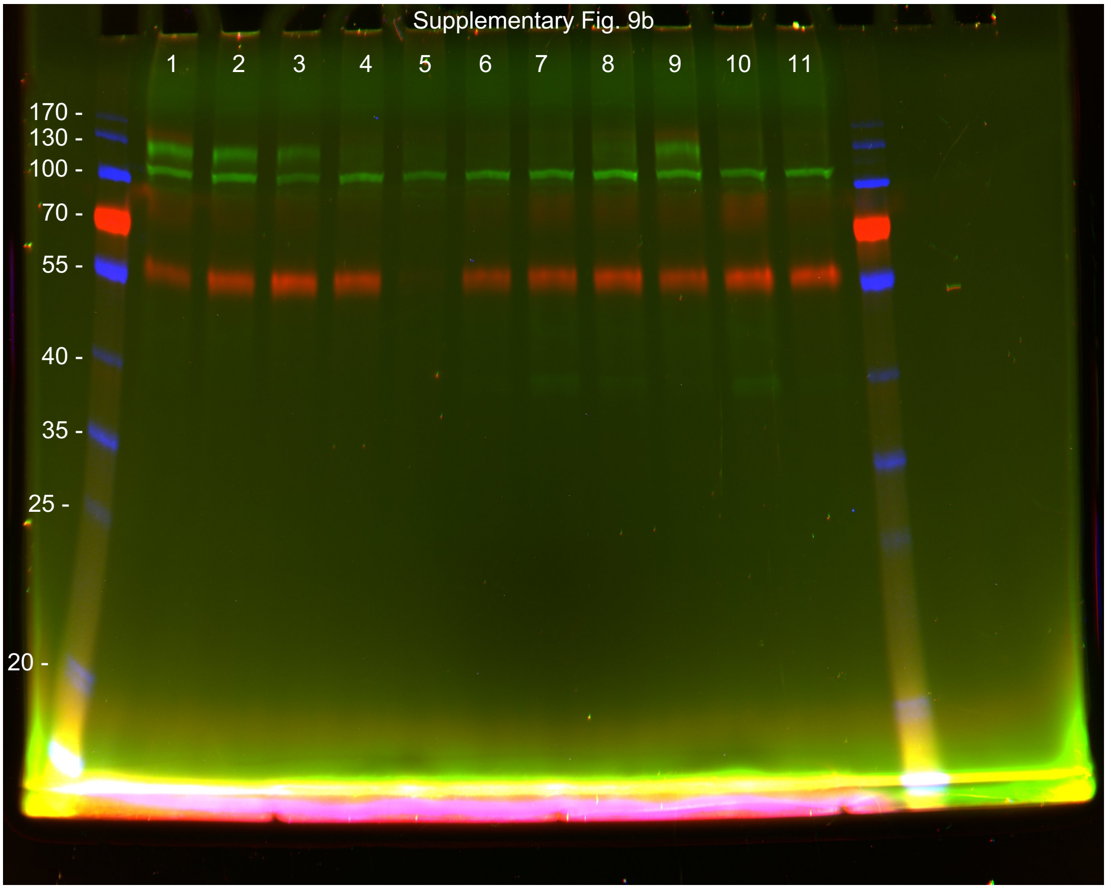

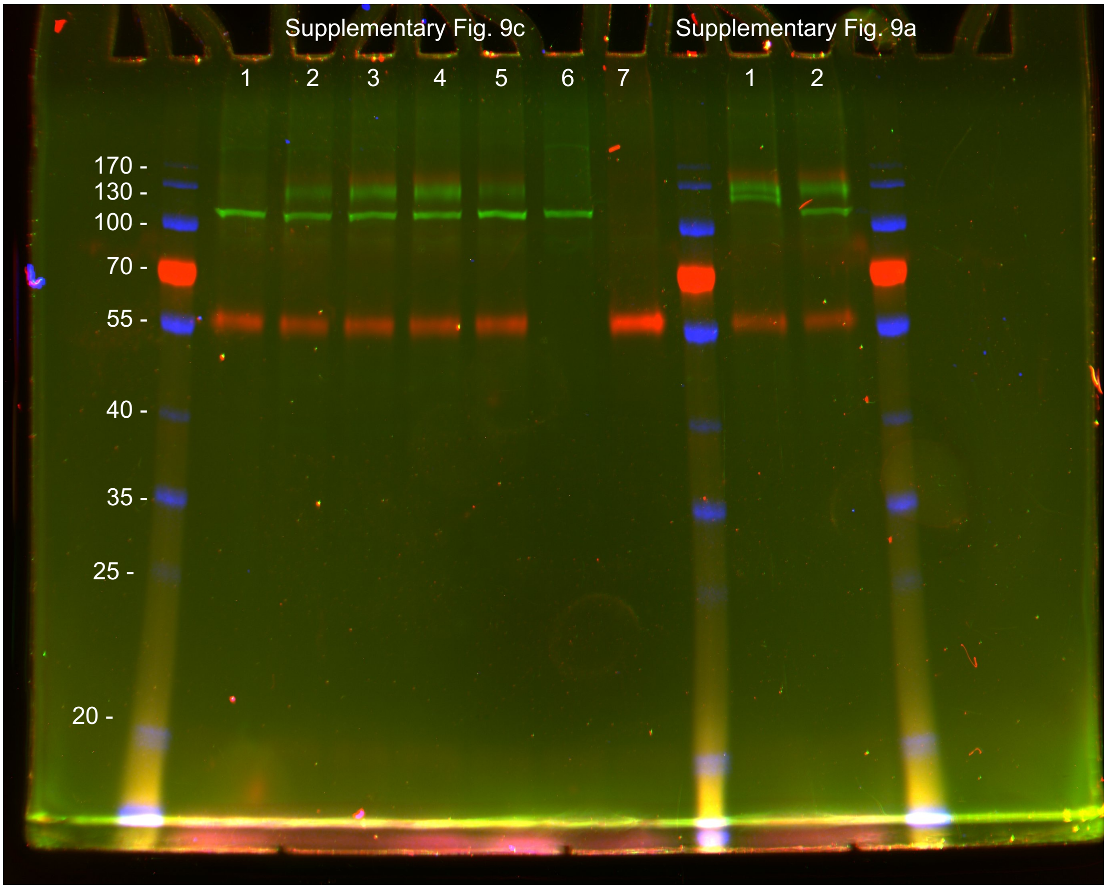

Supplementary Fig. 10: uncropped images
(Cropped images of the same gels are also shown in Figs. 5 and 8)

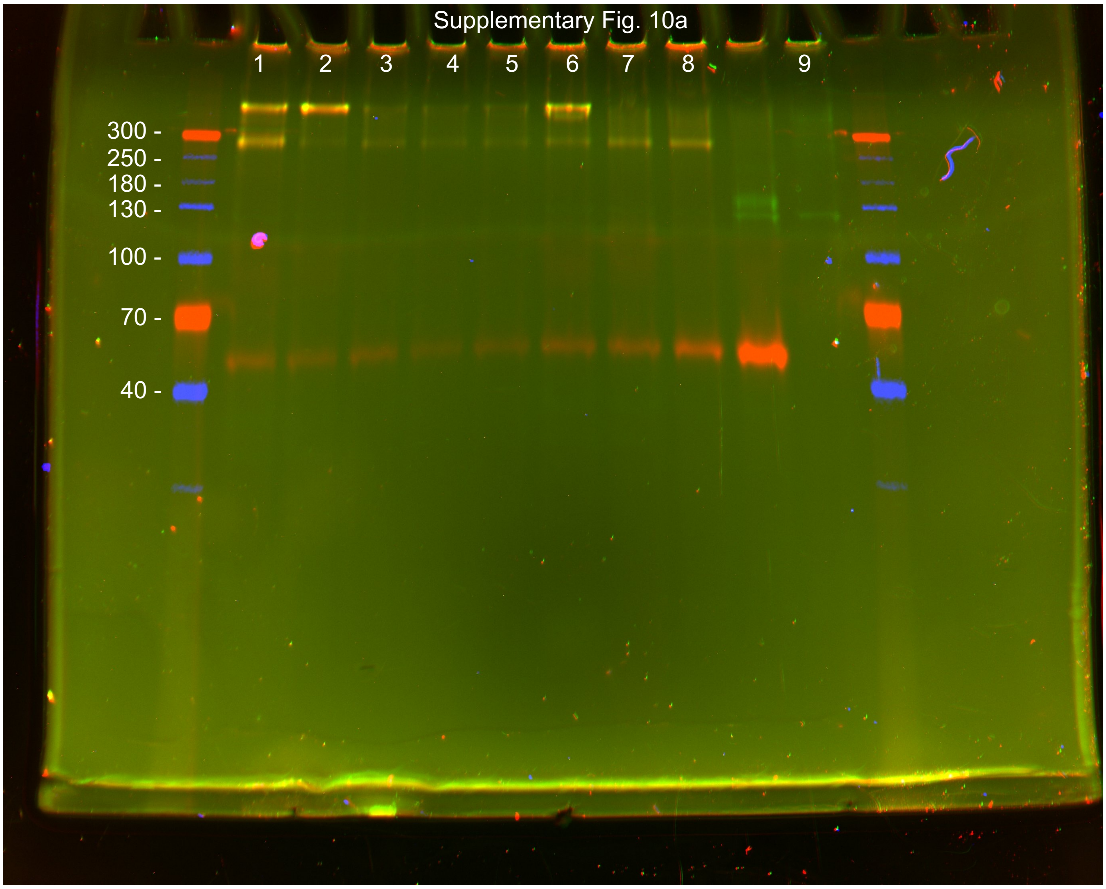

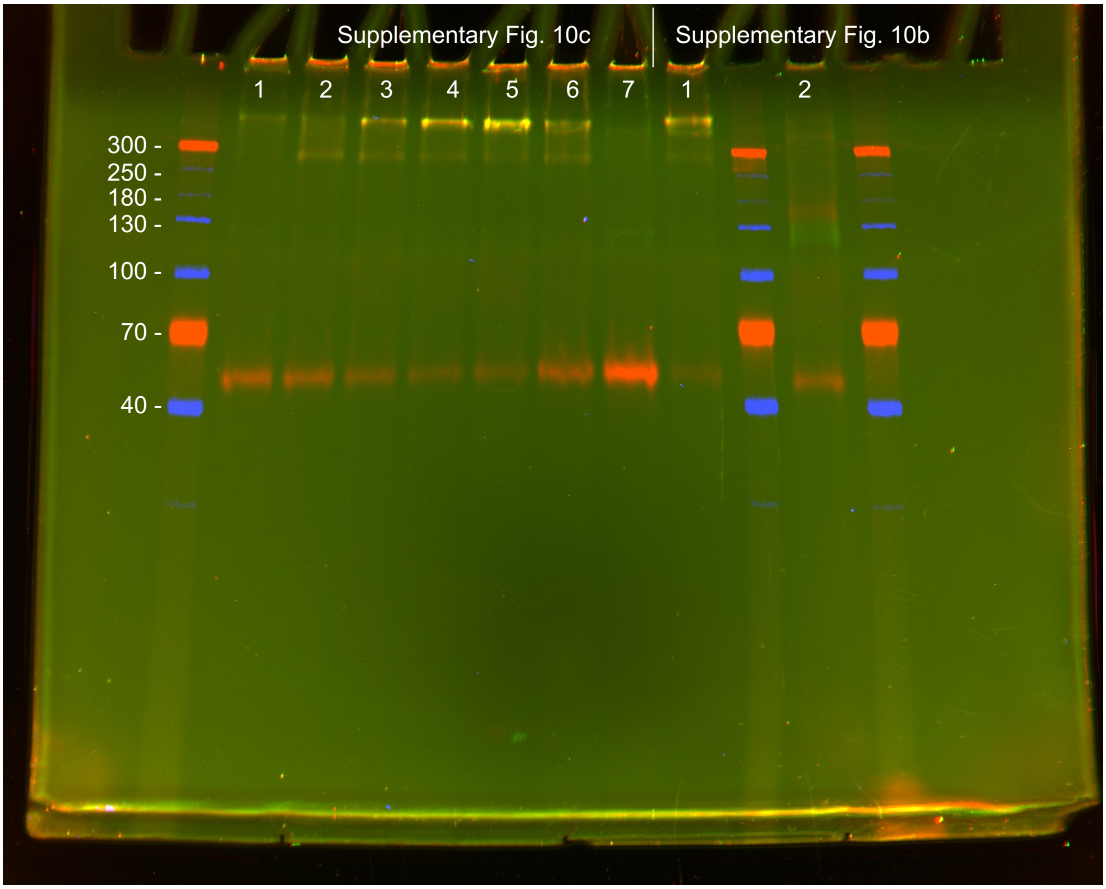

Supplementary Fig. 11a: uncropped images

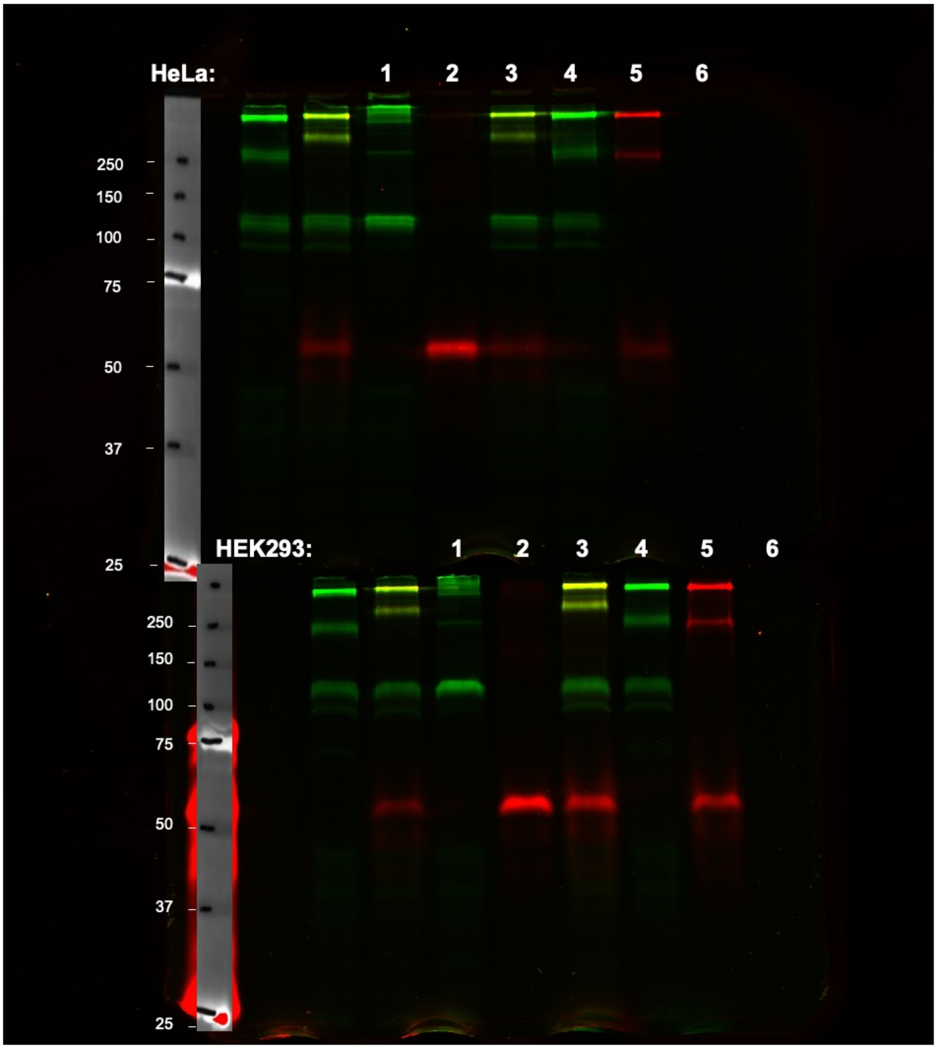

Data for Supplementary Fig. 11b: Uptake of L-[^{14}C]Ornithine							
Lane	Replicate	1	2	3	4	5	6
pEG-rBAT		GFP-rBAT	-	GFP-rBAT	GFP-rBAT	rBAT (no tag)	rBAT (no tag)
pEG-b$^{0,+}$AT		-	mCherry-b$^{0,+}$AT	mCherry-b$^{0,+}$AT	b$^{0,+}$AT (no tag)	mCherry-b$^{0,+}$AT	b$^{0,+}$AT (no tag)
Uptake (% control)	1	38.79	34.29	69.16	74.94	81.6	98.83
	2	41.06	40.07	98.66	91.24	97.9	99.71
	3	43.17	41.65	103.86	110.28	98.54	101.46
	mean	41.01	38.67	90.56	92.15	92.68	100.00
	SEM	1.26	2.24	10.80	10.21	5.54	0.77

Supplementary Fig. 12: uncropped images

Supplementary Fig. 12a: rBAT-WT

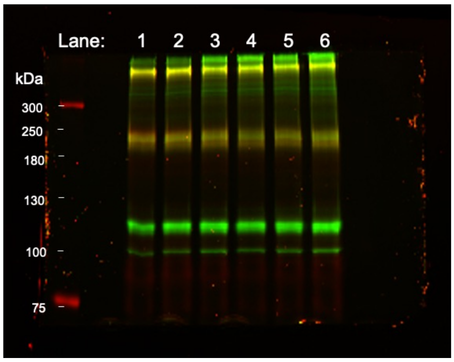

Supplementary Fig. 12b: rBAT-N214A

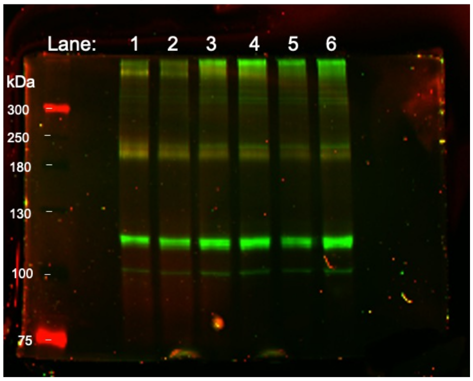

Supplementary Fig. 12c: rBAT-D284A

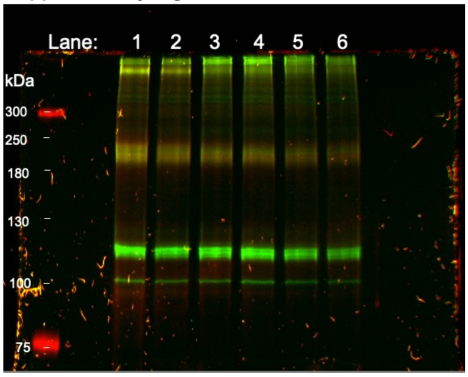

Supplementary Fig. 12d: rBAT-E321A

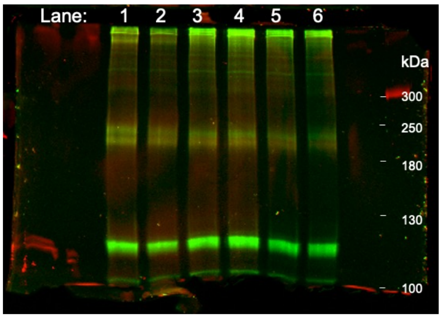

Supplementary Fig. 12e: rBAT-E321K

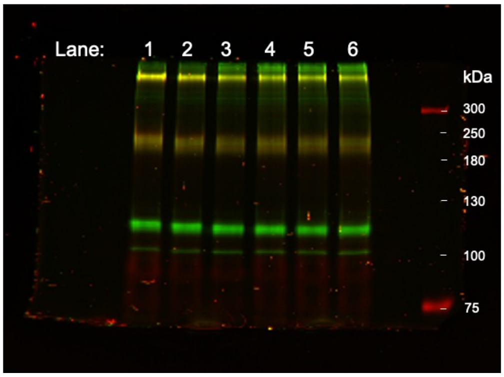

Supplementary Fig. 12f: CD98hc-LAT1

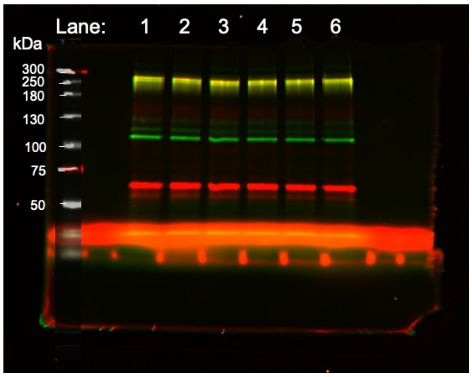

Supplement: Supplementary file 1 — Supplementary Information [file 41467_2022_30293_MOESM1_ESM.pdf]
