# Supplementary material for: Ca2+-mediated higher-order assembly of heterodimers in amino acid transport system b0,+ biogenesis and cystinuria
Source: Nat Commun. 2022 May 16;13:2708. doi: 10.1038/s41467-022-30293-9 (PMC9110406; doi:10.1038/s41467-022-30293-9)
Supplement: Supplementary file 3 — Description of Additional Supplementary Files [file 41467_2022_30293_MOESM3_ESM.pdf]

Description of Additional Supplementary Files

File name: Supplementary Movie 1

Description: Morphing of ovine $b^{0,+}$AT-rBAT super-dimer maps along the first principal component after multibody refinement
