# Supplementary material for: Ca2+-mediated higher-order assembly of heterodimers in amino acid transport system b0,+ biogenesis and cystinuria
Source: Nat Commun. 2022 May 16;13:2708. doi: 10.1038/s41467-022-30293-9 (PMC9110406; doi:10.1038/s41467-022-30293-9)
Supplement: Supplementary file 5 — Reporting Summary [file 41467_2022_30293_MOESM5_ESM.pdf]

Corresponding author(s): Yongchan Lee
Last updated by author(s): Feb 24, 2022

Reporting Summary

Nature Portfolio wishes to improve the reproducibility of the work that we publish. This form provides structure for consistency and transparency in reporting. For further information on Nature Portfolio policies, see our Editorial Policies and the Editorial Policy Checklist.

Statistics

For all statistical analyses, confirm that the following items are present in the figure legend, table legend, main text, or Methods section.

n/a	Confirmed	
☐	☒	The exact sample size (*n*) for each experimental group/condition, given as a discrete number and unit of measurement
☐	☒	A statement on whether measurements were taken from distinct samples or whether the same sample was measured repeatedly
☒	☐	The statistical test(s) used AND whether they are one- or two-sided<br>*Only common tests should be described solely by name; describe more complex techniques in the Methods section.*
☒	☐	A description of all covariates tested
☒	☐	A description of any assumptions or corrections, such as tests of normality and adjustment for multiple comparisons
☐	☒	A full description of the statistical parameters including central tendency (e.g. means) or other basic estimates (e.g. regression coefficient) AND variation (e.g. standard deviation) or associated estimates of uncertainty (e.g. confidence intervals)
☒	☐	For null hypothesis testing, the test statistic (e.g. *F*, *t*, *r*) with confidence intervals, effect sizes, degrees of freedom and *P* value noted<br>*Give P values as exact values whenever suitable.*
☐	☒	For Bayesian analysis, information on the choice of priors and Markov chain Monte Carlo settings
☒	☐	For hierarchical and complex designs, identification of the appropriate level for tests and full reporting of outcomes
☒	☐	Estimates of effect sizes (e.g. Cohen's *d*, Pearson's *r*), indicating how they were calculated

Our web collection on statistics for biologists contains articles on many of the points above.

Software and code

Policy information about availability of computer code

Data collection	EPU 2.4 and 2.5
Data analysis	RELION 3.0 and 3.1, MotionCor2, Gctf4, MODELLER 9.22, COOT 0.8.9, PHENIX 1.16-dev3689, UCSF ChimeraX 0.9, 1.1 and 1.2, APBS 3.0, Clustal Omega 1.2.2, ESPript 3, ImageJ 1.51, GraphPad Prism 8.4 , Fityk 1.3.1, Image Lab 6.0.1.

For manuscripts utilizing custom algorithms or software that are central to the research but not yet described in published literature, software must be made available to editors and reviewers. We strongly encourage code deposition in a community repository (e.g. GitHub). See the Nature Portfolio guidelines for submitting code & software for further information.

Data

Policy information about availability of data

All manuscripts must include a data availability statement. This statement should provide the following information, where applicable:

- Accession codes, unique identifiers, or web links for publicly available datasets
- A description of any restrictions on data availability
- For clinical datasets or third party data, please ensure that the statement adheres to our policy

The atomic coordinates have been deposited to Protein Data Bank under accession numbers 7NF6 (b0,+AT–rBAT heterodimer), 7NF7 (rBAT ectodomain) and 7NF8 (full complex composite model). Cryo-EM maps have been deposited to Electron Microscopy Data Bank under accession numbers EMD-12296 (b0,+AT–rBAT heterodimer), EMD-12297 (rBAT ectodomain) and EMD-12298 (full complex). Unprocessed gel pictures and raw data for all graphs were included in the Source Data file, provided with this paper. All other data will be available upon request.

Field-specific reporting

Please select the one below that is the best fit for your research. If you are not sure, read the appropriate sections before making your selection.

☒ Life sciences ☐ Behavioural & social sciences ☐ Ecological, evolutionary & environmental sciences

For a reference copy of the document with all sections, see nature.com/documents/nr-reporting-summary-flat.pdf

Life sciences study design

All studies must disclose on these points even when the disclosure is negative.

Sample size	No statistical methods were used to predetermine the sample size. The criteria for sample size is based on the widely accepted criteria used in previous publications in which similar types of experiments were proceeded.<br>For example, uptake experiments are usually performed in 3-4 experimental replicates which show sufficient sample numbers for calculation of standard error or standard deviation and statistical significance (although the statistical significance was not applied in this study).
Data exclusions	Data exclusion was performed during 2D and 3D image classification to remove particle images that did not contribute to high-resolution reconstructions, as is commonly performed in single-particle cryo-EM analyses.
Replication	All experiments were performed at least 3 times independently from different cell passages and showed similar results. Displayed Figures were derived from representative data of reproducible experiments. Transport assays in the transfected cells were performed in 3-4 technical replicates. The experiments were repeated 2-3 times independently using different cell passages and showed similar results.
Randomization	No specific randomization method was proceeded due to clinical or animal non-relevance. The experiments in this study utilize cell lines which passed the quality control from the manufacturers and stably exhibit normal morphology and growth along the study. The cells were self-randomly sampled in all treatment conditions, and all cell samples displayed normal morphology and similar cell numbers during all treatment processes.
Blinding	No particular blinding test was applied due to clinical or animal non-relevance. This study utilizes cell lines which exhibited stable conditions along the study. However, cell sampling for all treatments can be considered as self blinded sampling and randomization.

Reporting for specific materials, systems and methods

We require information from authors about some types of materials, experimental systems and methods used in many studies. Here, indicate whether each material, system or method listed is relevant to your study. If you are not sure if a list item applies to your research, read the appropriate section before selecting a response.

Materials & experimental systems

n/a	Involved in the study	
☐	☒	Antibodies
☐	☒	Eukaryotic cell lines
☒	☐	Palaeontology and archaeology
☒	☐	Animals and other organisms
☒	☐	Human research participants
☒	☐	Clinical data
☒	☐	Dual use research of concern

Methods

n/a	Involved in the study	
☒	☐	ChIP-seq
☒	☐	Flow cytometry
☒	☐	MRI-based neuroimaging

Antibodies

Antibodies used	The GFP nanobody "N8his-GFPenhancer-GGGGS4-LaG16" (self production from the given clone from Dr. Motoyuki Hattori. Ref. 76; AddGene plasmid #140442)<br>The GFP nanobody "enhancer" (expressed from an in-house made plasmid based on a published work: U. Rothbauer, K. Zolghadr, S. Muyldermans, A. Schepers, C. M. Cardoso, H. Leonhardt, A versatile nanotrap for biochemical and functional studies with fluorescent fusion proteins. Mol Cell Proteomics. 7, 282–289 (2008))<br>Anti-PDI (C81H6 lot. 3; Cell Signaling#3501S)<br>Anti-RCAS1 (D2B6N lot. 4; Cell Signaling#11290S)
Validation	Anti-PDI (rabbit mAb reacts to human protein) and anti-RCAS1 (rabbit mAb reacts to human protein) antibodies were in-house validated by Cell Signaling company. The antibodies are specific and consistent from lot-to-lot and applicable to Immunoflorescent imaging (IF).<br><br>It is noted that the authors also performed validation test prior to use anti-PDI and anti-RCAS1 in this study. Anti-PDI was co-stained with anti-GRP78 (mouse mAb: Invitrogen#MA5-27686 lot.WG3318092) while anti-RCAS1 was co-stained with anti-58K (mouse mAb:

Novus#NB600-412SS lot. A-6) in HeLa cells (host cells in this study). Both experiments confirm the validation of anti-PDI and anti-RCAS1 as ER and Golgi markers, respectively.

Eukaryotic cell lines

Policy information about cell lines

Cell line source(s)	HEK293S GnTI- (ATCC, catalog number CRL-3022), HEK-293 (ATCC, catalog number CRL-1573) and HeLa cells (ATCC, catalog number CCL-2; or CLS Cell Lines Services, catalog number 300194)
Authentication	All cell lines have been authenticated by suppliers. The cell lines used in all biochemical experiments were authenticated by STR profiling from the manufacturers and displayed appropriate morphologies and growth during all experimental procedures.
Mycoplasma contamination	Cell lines were originally tested for Mycoplasma contamination from the manufacturers. However, they were not tested during the study.
Commonly misidentified lines (See ICLAC register)	The above cell lines are not listed in the commonly misidentified cell lines.
